# The correlates of HIV testing and impacts on sexual behavior: evidence from a life history study of young people in Kisumu, Kenya

**DOI:** 10.1186/1471-2458-10-412

**Published:** 2010-07-13

**Authors:** Caroline W Kabiru, Nancy Luke, Chimaraoke O Izugbara, Eliya M Zulu

**Affiliations:** 1 African Population and Health Research Center, 2nd Floor Shelter Afrique Centre, P. O. Box 10787-00100, Nairobi, Kenya; 2 Department of Sociology, Population Studies and Training Center, Brown University, Box 1916, Providence, Rhode Island, 02912 USA; 3 African Institute for Development Policy (AFIDEP), P. O. Box 14688-00800, Nairobi, Kenya

## Abstract

**Background:**

HIV counseling and testing is considered an important component of HIV prevention and treatment. This paper examines the characteristics of young males and females at the time of first reported HIV test, including the influence of recent sexual partnerships, and investigates how HIV testing and the cumulative number of tests are associated with sexual behaviors within six months of testing.

**Methods:**

The study uses data from a random sample of youth aged 18-24 years living in Kisumu, Kenya, who were interviewed using a 10-year retrospective life history calendar. Cox regression models were used to examine the correlates of the timing of first HIV test. Variance-correction models for unordered repeated events were employed to examine whether having an HIV test in the previous six months and the cumulative number of tests predict unsafe sexual practices in a given month.

**Results:**

Sixty-four percent of females and 55% of males reported at least one HIV test in the last 10 years and 40% of females were pregnant the month of first test. Significant correlates of first HIV test included marital aspirations among non-pregnant females, unprotected sex in the previous six months among pregnant females, and concurrency in the previous six months among males. Having a recent HIV test was associated with a decreased likelihood of unprotected sex among ever-pregnant females, an increased likelihood of unprotected sex and "risky" sexual partnerships among never-pregnant females, and an increased likelihood of concurrency among males. Repeated HIV testing was associated with a lower likelihood of concurrency among males and involvement in "risky" sexual partnerships among males and never-pregnant females.

**Conclusions:**

The high rate of pregnancy at first test suggests that promotion of HIV testing as part of prevention of mother-to-child transmission is gaining success. Further research is warranted to examine how and why behavior change is influenced by client- versus provider-initiated testing. The influence of different sexual partnership variables for males and females suggests that interventions to assess risk and promote testing should be gender- and relationship-specific. The findings also suggest that encouraging repeat or routine testing could potentially increase the uptake of safer sexual behaviors.

## Background

HIV counseling and testing is widely considered an important and cost-effective component of HIV prevention and treatment [1,2]. The evidence of the impact of HIV counseling and testing on subsequent high-risk sexual behavior has been mixed, however. While several studies have found significant declines in the reporting of multiple partnerships and unprotected sex, for example [3-5], other studies have found little or negative effects of testing on behavior change [6,7].

Epidemiological data from the 2007 Kenya AIDS Indicator Survey (KAIS) indicate an HIV prevalence rate of 7.1% among adults aged 15-64 years [8]. Geographically, Nyanza Province, whose provincial capital, Kisumu city, is the setting for the present study, is the most heavily affected region in Kenya with an adult prevalence rate of 14.9%. The principal mode of viral transmission in Kenya is heterosexual contact [9]. Voluntary counseling and testing (VCT) is an important component of preventive efforts [10]. Through collaborative efforts by the government and other primary stakeholders, the number of VCT facilities in the country increased from three to over 500 between 2000 and 2005 [10]. The primary model for HIV testing has been client-initiated; however, provider-initiated testing is becoming more common through prevention of mother-to-child transmission (PMTCT) services and other health care programs [10]. Increased accessibility of testing facilities combined with higher demand has led to greater uptake of HIV testing services in Kenya. In 2003, 14% of adults had ever been tested, and by 2007, 37% reported at least one HIV test with testing rates higher in urban areas [8].

Throughout sub-Saharan Africa, HIV prevalence rates are generally higher among female adolescents and young adults [11-16]. Among Kenyan youth aged 15-24, HIV prevalence is estimated at 5.6% for females and 1.4% for males [8]. Rates of HIV infection are much higher in urban Kisumu with earlier estimates showing that almost 12% of females and 4% of males aged 15-19 years are HIV infected. Comparative figures for females and males aged 20-24 years are 40% and 15%, respectively [17,18]. The high risk of infection could spur increased uptake in testing among young people in Kisumu, however population estimates of HIV testing are unavailable. Data from the 2007 KAIS indicate that nationally, HIV testing rates are highest among females aged 20-24 years. In this age group, 66% of females who are sexually experienced have had an HIV test. Comparative figures for sexually experienced females aged 15-19 years, males 15-19 years, and males 20-24 years are 46%, 15%, and 32% respectively. Although these numbers reflect a significant increase in testing rates, the uptake of HIV testing remains below the government's 2010 goal of 80% coverage [8].

The exceptionally high rates of HIV infection among youth in Kisumu underscore the need for effective preventive and treatment programs tailored to young people. Efforts to develop such programs are often curtailed by lack of empirical evidence showing linkages between knowledge, perception of risk, adoption of preventive practices, indulgence in unsafe sexual practices, and cost effectiveness of various intervention programs. In particular, despite a substantial number of studies assessing HIV testing and its effects on high-risk behavior in general populations, there is a dearth of studies examining the correlates and impacts of HIV testing among adolescents and young adults [19]. With notable exceptions [15,16], many studies that have assessed HIV testing behavior among adolescents [20,21] have been conducted in western countries among high-risk youth or among sexually active adolescents in areas with much lower HIV prevalence rates than countries in many parts of sub-Saharan Africa. Where it exists, research has focused on correlates of HIV testing [20,21] with less attention to its impact.

Existing research on the correlates of HIV testing among youth--and among adult populations in general--is limited in several respects. First, most studies rely on current characteristics to predict the likelihood of ever having an HIV test [22,23]. Current characteristics may be poor predictors of past behavior, and more detailed information on respondents' characteristics and behaviors at the timing of testing would be more relevant. Second, most studies tend to focus on socio-demographic or cognitive factors, such as low perceived risk of infection or fears about knowing the results, as determinants of testing behavior [8,16,21]. Little attention has been paid to the social context surrounding testing decisions, particularly the potential importance of individuals' sexual histories and marital aspirations. The types of partnerships individuals engage in, as well as marital aspirations, infidelity, or unsafe sexual behaviors within them, may trigger the decision to test. With respect to research on the impact of HIV testing on behavior change, most studies have evaluated testing that has been exclusively provided through interventions [3,4,24,25], and it is, therefore, unclear whether the same effects would be observed in the general population. Finally, because few studies adopt a life-course approach and record individuals' HIV testing histories, it is not possible to examine both the immediate as well as cumulative effects of multiple HIV tests on subsequent sexual behavior.

This paper uses unique life history data collected from a population-based sample of youth aged 18-24 years in Kisumu, Kenya's third largest city, to examine HIV testing behavior in this setting of high HIV prevalence. These data come from a larger study whose overall aim was to assess the quality of sexual behavior data collected using a new survey instrument, the Relationship History Calendar (RHC). The analysis has three main aims. First, we describe the characteristics of young males and females at the time of first reported HIV test to gain greater insight into factors associated with testing. Second, we examine the correlates of the timing of first HIV test, paying particular attention to the influence of sexual partnership characteristics in the six months prior to the test. Finally, we examine how recent HIV testing and the cumulative number of tests influence sexual behavior six months after testing to explore the immediate influence of HIV counseling and testing on sexual behavior, and if testing has a greater impact for those who repeat test.

## Methods

### Study description

Data were collected in June to July 2007 under the *Urban Life among Youth in Kisumu *study conducted by a team of researchers from the African Population and Health Research Center (APHRC), Brown University, and McGill University. The RHC was designed to collect detailed, 10-year retrospective information on respondents' schooling, residence, sexual partnerships, and HIV testing histories. These data allow us to explore the correlates of HIV testing at the time of and immediately prior to testing as well as how individuals' HIV testing history affects subsequent sexual behaviors.

The study sample was drawn by contacting every other household in 45 randomly selected urban enumeration areas (EAs) out of approximately 400 EAs within Kisumu city limits. Men and women aged 18 to 24 years in the selected households were eligible to be interviewed; if more than one eligible person resided in a household, one was randomly selected. Given the overall aim of the larger study, selected respondents were randomly assigned to receive either the RHC or a standard sexual behavior questionnaire similar to those used in large-scale surveys, including the Demographic and Health Surveys [9]. Both the RHC and the standard questionnaire were administered face-to-face by trained interviewers. The present analyses use data from the 601 RHC respondents. Written informed consent was obtained from each respondent at the beginning of the interview, and the respondents received approximately US$2.80 at the completion of the interview as compensation for their time. Ethical clearance was granted by the Kenya Medical Research Institute (KEMRI), the Kenyan Ministry of Science and Technology, and Brown and McGill Universities.

### Study instruments

The RHC is a 10-year retrospective life history calendar that gathers detailed monthly information on life course domains, including sociodemographic history (residence, schooling, and employment), sexual and non-sexual partnerships, and the respondent's HIV testing history. Within each relationship, information was recorded on partnership attributes (e.g., relationship type, aspirations for marriage, coital frequency and condom use, perceived infidelity of the partner, and pregnancies and outcomes). To enhance recall of the occurrence and timing of each partnership and its characteristics, respondents referenced the dates of personal (e.g., the birth of a child, completion of primary school) and public (e.g., the 1998 US Embassy Bombing in Nairobi) landmark events as well as the timing and sequencing of their schooling, residence, and other partnership trajectories on the calendar. A separate module of the questionnaire obtained data on gender, ethnicity, and religion. Several studies [26-31] have found that life history calendars reduce recall error and significantly increase data reliability. For example, Freedman and colleagues [28] compared nine-year retrospective data collected from a sample of 23-year-old Americans and noted significant concordance with data collected from the same group five years prior to the administration of the calendar. In addition, the RHC adopts a conversational style that is similar to an in-depth interview and has a flexible structure that allows respondents to discuss their relationships in a sequence that is comfortable to them. Consequently, young people may be more willing to openly discuss sensitive matters such as sexual behavior. Indeed, the larger study found that the RHC interview fostered substantially higher levels of rapport and respondent enjoyment than the standard face-to-face interview. These findings suggest that the RHC reduced social desirability bias, and, overall, improved reporting of multiple measures of sexual behavior in comparison to the standard questionnaire [32].

### Measures

#### HIV testing

Respondents reported monthly retrospective information, including their HIV testing histories, beginning in 1998 when they were aged 8 to 14, and continuing until the month of the survey in 2007. We do not know if respondents tested for HIV before 1998; however, given that testing facilities were very few in Kenya [10] and the respondents were generally quite young, it is likely that the RHC has captured all tests that the vast majority of the respondents had in their lives.

The monthly calendar data allow us to use person-months as the unit of analysis, and all variables are constructed for each person-month on the calendar. For the descriptive and survival analyses of HIV testing, we constructed a dichotomous variable for each month indicating whether or not a respondent reported an HIV test and received the test results. Respondents did not receive their results in only 1.5% of all reported testing occurrences. For the survival analyses, we designate the exposure period as age 14 and above, so all respondents' histories begin at the same chronological age. Only five respondents reported their first test before age 14, and these tests are left censored in the survival analyses.

To assess the impact of having one or more tests on subsequent sexual behavior, we created a dichotomous variable for each month indicating whether or not the respondent had an HIV test in the previous six months as well as a continuous variable indicating the cumulative number of tests since age 14 including that month and used these as our key independent variables.

#### Additional variables

We included individual and sexual partnership history characteristics as independent variables in the analyses. Variables for individual characteristics were constructed as the present value for each month in the calendar (and are therefore time-varying), with the exception of religion and ethnicity, which take on the same value for each month of the calendar. Individual characteristics included respondents' age, current schooling status (in- versus out-of-school), highest completed level of schooling (no schooling or primary, secondary, and college/university). Religion comprised five categories (Catholic, Protestant, Pentecostal, African Traditional, and "Other"). We created three categories for ethnicity: Luo, Luhya, which are the two major ethnic groups in Kisumu City, and "Other". The RHC recorded monthly information on occupation and income, which allowed us to create a dichotomous variable indicating whether a respondent earned any income in a particular month or not. We also considered whether a respondent was living in an urban or rural area in each month. We created a pregnancy status (self or partner) variable for each respondent for each calendar month.

Historically, marriage in sub-Saharan Africa was a union between families; thus, the family played an important role in the choice of a marital partner [33]. However, marriage is increasingly seen as a union between two individuals and individual marital aspirations may have an important bearing on partnership behaviors including HIV testing. Thus, for each month, we created a trichotomous variable indicating if the respondent was married, unmarried and wished to marry any of his or her partners, or unmarried and did not wish to marry any of his or her partners.

HIV testing may also be driven by the characteristics of one's partners. To test the hypotheses that previous behavior affects the decision to test, we constructed partnership characteristics pertaining to the six-month period prior to each calendar month (and they are therefore time-varying). All the partnership characteristics are dichotomous *yes *or *no *variables. Individuals may be more likely to get tested if they perceive that one or more of their partners are at high risk of HIV infection. We therefore constructed a variable for engaging in a "risky" sexual partnership, defined as a partnership with a casual partner, commercial sex worker or client, "one-night stand", or a stranger in the previous six months. Individuals who know or suspect their partners to have other sexual relationships may believe they are at greater risk of infection. Thus, we constructed a variable designating whether the respondent knew or suspected any of their partners to have marital or non-marital partners other than him/herself in the previous six months. Finally, as the decision to test could be driven by unsafe sexual behaviors within recent partnerships, we assessed whether the respondent had unprotected sex in any partnership or concurrent sexual partners in the previous six months. Unprotected sex in a given month was constructed as a dichotomous variable indicating whether or not condoms were *always *used with *all *sexual partners. Concurrency was defined as having two or more sexual partners in any particular month.

To test the hypothesis that recent HIV testing history and the cumulative number of tests affect subsequent sexual behavior, we created dichotomous dependent variables that included having a "risky" sexual partner, concurrent partnerships, or unprotected sex in the month.

### Data analyses

The first part of our analysis presents descriptive statistics with respect to HIV testing. Similar to previous studies of the determinants of ever testing for HIV, we examine differences in individual characteristics between those who report at least one HIV test in the 10-year period from 1998-2007 and those who did not report an HIV test. Taking advantage of the monthly information provided on the calendar, we next examine individual characteristics during the month of respondents' first HIV test and sexual partnership history characteristics pertaining to the six months prior to the first test for males and females.

The second part of the analysis uses Cox regression models to examine the correlates of the timing of the first HIV test. As noted above, the exposure period begins at age 14 for all respondents. The time to event in the regression models is defined as time in months from age 14 to the first HIV test. Two hundred and forty-five (245) respondents are right-censored because they report no HIV test by the time of survey. The remaining respondents contribute a total of 356 HIV testing episodes. Data from males and females are analyzed separately to explore differences by gender. About 40% of tested women were pregnant during their first HIV test. Given potential differences in determinants of testing among pregnant versus non-pregnant females, we present results separately for females based on pregnancy status at first test. Hazard ratios indicating the relative likelihood of reporting the first HIV test in a given month based on the independent variables and 95% confidence intervals are reported.

The final analyses use Cox regression models that account for repeated unordered events using robust standard errors clustered by respondent [34] to examine whether having an HIV test in the previous six months and the cumulative number of tests predict unsafe sexual practices, including concurrent sexual partnerships, unprotected sex, or engaging in a "risky" partnership, in a given month. We control for individual characteristics in all the models. Because HIV testing at the time of pregnancy is likely provider-driven rather than client-initiated, we run separate models for never-pregnant and ever-pregnant women in an attempt to isolate the relationship between client-initiated or provider-driven testing and subsequent sexual activity for young women.

## Results

Table 1 summarizes respondents' characteristics by gender and testing status. Overall 356 male and female respondents (59%) reported at least one HIV test. The number of tests reported ranged from 1 to 18. Over 15% of those who had ever been tested reported three or more tests. A greater proportion of females (64%) than males (55%) reported an HIV test. Respondents who reported that they have ever been tested for HIV were significantly older than their never-tested peers. Just under one-third of females were married and of these, four-fifths had been tested for HIV (not shown). The sample distribution by highest level of education shows that a significantly greater proportion of males who had ever been tested had secondary or higher education than their never tested counterparts. Sixty-six percent of males who were in school at the time of the survey had been tested compared to 50% of those not in school (not shown). The sample distributions for earnings, ethnicity, and religion did not differ significantly by testing status for both males and females.

**Table 1:** Descriptive characteristics of respondents (at month of interview), by gender and testing status

	Female				Male			
		
	Never tested	Ever tested		Total	Never tested	Ever tested		Total
	101	181		282	144	175		319
	35.8%	64.2%	p-value		45.1%	54.9%	p-value	
Mean age in years (standard deviation)	20.2	20.8	*	20.6	20.4	21.0	**	20.7
	(1.71)	(1.92)		(1.86)	(1.82)	(1.90)		(1.88)
								
% Married	15.8	34.3	**	27.7	7.6	8.6		8.2
								
Highest level of education (%)								
None/Primary	31.7	38.1		35.8	34.7	18.9	**	26.0
Secondary	64.4	58.0		60.3	61.1	74.9		68.7
Some college	4.0	3.9		3.9	4.2	6.3		5.3
								
% in school	19.8	18.8		19.2	22.9	36.0	*	30.1
								
% earning an income	22.8	31.5		28.4	42.4	44.0		43.3
								
Ethnicity (%)								
Luo	67.3	72.9		70.9	72.2	78.9		75.9
Luhya	18.8	19.3		19.2	11.8	10.3		11.0
Other	13.9	7.7		9.9	16.0	10.9		13.2
								
Religion (%)								
Catholics	24.0	27.1		26.0	21.5	25.1		23.5
Protestants	47.0	34.8		39.2	47.9	41.7		44.5
Pentecostal	18.0	22.1		20.6	13.2	16.6		15.1
African/Traditional	9.0	9.4		9.3	11.1	6.9		8.8
Muslims/Other/none	2.0	6.6		5.0	6.3	9.7		8.2
								
Median number of HIV tests since age 14 (Range)		1 (1-10)				1 (1-18)		
								
Number of HIV tests since age 14 (%)								
One		56.9				62.3		
Two		27.6				20.6		
Three		7.7				9.1		
Four or more		7.7				8.0		

*p < 0.05, **p < 0.01

Table 2 summarizes respondent characteristics at first HIV test by gender and pregnancy status for females. The mean age at first test was about 19 years for all groups. Not surprisingly given the links between fertility and educational attainment in previous studies in sub-Saharan Africa [35], almost three times as many pregnant females (64%) as their non-pregnant peers (20%) had less than secondary education when tested for the first time. With respect to partnership characteristics at the time of first HIV test, about 50% of pregnant females were married at the time of their first test, while only 15% were married among the non-pregnant females. A larger proportion of males (13%) than females (6% for the full sample) reported that they had a "risky" sexual partner in the six months preceding the first test. A larger percentage of males (10%) than females (3% for the full sample) reported concurrent sexual partnerships during the six months prior to their first test, while a larger percentage of females (58% of the full sample) than males (28%) reported unprotected sex during this time period. Because unprotected sex is more common in marriage (results not shown), the large proportion of females who reported unprotected sexual intercourse may be heavily influenced by the large number of married females in the sample. In addition, unprotected sex is a precondition for pregnancy, and thus a very large percentage of women who were pregnant at first test (over 90%) reported having unprotected sex during the six months prior to the test. Interestingly, there was no significant difference between males and females with respect to whether the respondent knew or suspected that his/her partner(s) had other partners in the six months before their first HIV test.

**Table 2:** Individual and partnership characteristics in the month of first HIV test, by gender and pregnancy status (for females)

	Female		Male
			
	Not pregnant	Pregnant at first test	
		
	108 (59.7%)	73 (40.3%)	175
*Individual Characteristics*			
Mean age in years (standard deviation)	19.0	18.6	19.1
	(2.20)	(2.03)	(2.18)
Median age (Years)	20.2	19.3	20.0
Highest level of education (%)			
None/Primary	20.4	64.4	21.1
Secondary	76.9	35.6	75.4
Some college	2.8	0.0	3.4
In school (%)	39.8	13.7	49.7
Earning an income (%)	22.2	17.8	37.1
Residing in an urban area (%)	91.6	91.8	92.0
Pregnant (partner) (%)	--	--	3.4
			
*Partnership Characteristics*			
Marital status and aspirations			
Not married; does not wish to marry any partner (%)	56.5	24.7	68.0
Not married; wishes to marry at least one partner (%)	28.7	26.0	26.9
Married (%)	14.8	49.3	5.1
6 months before first test:			
Had a "risky" partner (%)	6.5	4.1	13.1
Partner had other partners (known/suspected) (%)	21.3	17.8	19.4
Had concurrent sexual partners (%)	2.8	4.1	9.7
Had unprotected sex (%)	36.1	90.4	28.0

To evaluate the correlates of the timing of the first HIV test, we employed Cox regression models (Table 3). Among non-pregnant females, we found that urban residence and marital aspirations were positively and significantly associated with HIV testing. Specifically, females were more likely to be tested if they were not married but wished to marry at least one of their partners compared to unmarried females who did not wish to marry any partner. Among females who were pregnant at their first HIV test, age and earning an income were both negatively associated with HIV testing. We postulate that these associations may indicate a greater propensity by older women and those who have some income to opt out of PMTCT-related testing. In addition, among pregnant women, those who engaged in unprotected sex in the six months preceding the test were 8.3 times more likely to report an HIV test, which may reflect the timing of pregnancy and antenatal care visits (and accompanying provider-initiated HIV testing) that occur in the first few months after pregnancy has been established. Among males, education, and urban residence were positively associated with the likelihood of testing and those who did not belong to the two predominant ethnic groups (Luo or Luhya) were significantly less likely to report an HIV test than Luos. Male respondents who had concurrent sexual partners in the last six months were 3.2 times more likely to report an HIV test than their peers who reported no concurrency. At least for males, therefore, testing might be prompted by unsafe sexual behavior in terms of concurrency.

**Table 3:** Hazard ratio estimates from Cox regression models of time since age 14 to first HIV test among Kisumu youth, by gender and pregnancy status (for females)

	Female				Males	
		
	Not pregnant at first test		Pregnant at first test			
		
Variables	Hazard Ratio	[95% CI]	Hazard Ratio	[95% CI]	Hazard Ratio	[95% CI]
*Sociodemographic characteristics*						
Age	0.83	[0.53,1.29]	0.40*	[0.17,0.96]	0.69	[0.44,1.07]
Highest level of education (ref. None/Primary)						
Secondary	1.47	[0.85,2.54]	1.45	[0.82,2.56]	1.86**	[1.25,2.77]
College	2.71	[0.75,9.85]	--		1.27	[0.50,3.24]
In school (ref. out of school)	0.95	[0.55,1.65]	0.48	[0.20,1.14]	1.08	[0.71,1.67]
						
Religion (ref. Protestant)						
Catholic	0.99	[0.62,1.59]	1.32	[0.71,2.44]	1.14	[0.78,1.68]
Pentecostal	0.9	[0.51,1.60]	1.61	[0.83,3.11]	1.26	[0.84,1.89]
African/Tradition	0.88	[0.38,2.08]	1.13	[0.55,2.32]	1.42	[0.69,2.92]
Other	0.98	[0.47,2.03]	1.18	[0.43,3.27]	1.57	[0.88,2.79]
Ethnicity (ref. Luo)						
Luhya	1.39	[0.80,2.44]	1.09	[0.51,2.34]	0.79	[0.46,1.37]
Others	0.68	[0.34,1.34]	0.69	[0.34,1.41]	0.61*	[0.39,0.96]
Earned income (ref. no income)	1.02	[0.57,1.80]	0.52*	[0.29,0.92]	1.12	[0.73,1.71]
Urban residence (ref. rural)	2.73**	[1.29,5.76]	1.84	[0.66,5.08]	2.10*	[1.16,3.81]
Pregnant partner (ref. non-pregnant partner)	--		--		0.99	[0.40,2.45]
						
*Partnership Variables*						
Not married; does not wish to marry any partner (ref)						
Not married; wishes to marry at least one partner	1.73*	[1.04,2.88]	0.64	[0.31,1.31]	1.05	[0.75,1.49]
Married	1.02	[0.48,2.20]	1.11	[0.52,2.34]	1.26	[0.43,3.75]
In the last six months:						
Had a "risky" partner	1.01	[0.41,2.47]	1.11	[0.33,3.72]	0.83	[0.49,1.42]
Had a partner with other partners	1.29	[0.74,2.25]	1.03	[0.47,2.25]	1.09	[0.72,1.66]
Had concurrent sexual partners	0.78	[0.25,2.39]	0.43	[0.11,1.65]	3.19**	[1.55,6.57]
Had unprotected sex	1.16	[0.69,1.95]	8.32**	[3.40,20.33]	0.93	[0.62,1.39]
						
Observations (person-months)	15167		4430		23653	
n	207		73		319	
Wald's Chi-square	32.62		66.18		44.01	

CI = Confidence Interval, Ref = Reference category

*p < 0.05, **p < 0.01

To assess the pattern of subsequent behavior change associated with testing, we examined the effect of having had an HIV test in the past six months and the cumulative number of tests since age 14 on the likelihood of reporting concurrent sexual partnerships, unprotected sex, and "risky" sexual partnerships in a given month (Table 4). Among never-pregnant females, recent testing was associated with increased unsafe sexual behaviors. Having an HIV test in the previous six months increased the likelihood of having unprotected sex by 1.6 times (marginally significant) and increased the likelihood of having a "risky" sexual partner by 3.5 times in any given month. However, larger numbers of HIV tests were associated with a lower likelihood of having a "risky" sexual partner (marginally significant) among these young women. Among ever-pregnant females, having an HIV test in the previous six months was associated with a 40% decrease in the likelihood of engaging in unprotected sex in any given month compared to those who did not test. Finally, males who reported an HIV test in the past six months were 3.2 times more likely to report concurrent sexual partnerships in a given month, while greater numbers of repeat tests were associated with a lower likelihood of having a concurrent sexual partnership or "risky" sexual partnership (marginally significant) six months later. Although we do not present the results here, we observed similar patterns in terms of the magnitude and significance levels for the HIV testing variable if we restricted the time of testing to the last 3 months, which suggest that the more immediate influence of HIV testing on subsequent sexual behaviors three months after testing is sustained for the period up to six months after testing.

**Table 4:** Hazard ratio estimates of involvement in sexual risk behavior from variance-correction models for repeated events, by gender and pregnancy status (for females)

	**Never pregnant females (N = 133)**		**Ever pregnant females (N = 147)**		**Male (N = 319)**	
			
	**Hazard Ratio**	**95% Confidence Interval**	**Hazard Ratio**	**95% Confidence Interval**	**Hazard Ratio**	**95% Confidence Interval**
	
Concurrent sexual partnerships						
Had an HIV test in last 6 months	0.69	[0.07,7.12]	1.67	[0.51,5.48]	3.18**	[1.51,6.72]
Cumulative number of HIV tests since age 14	0.59	[0.30,1.14]	0.53	[0.16,1.69]	0.57**	[0.40,0.82]
						
Unprotected sex						
Had an HIV test in last 6 months	1.64^†^	[0.94,2.83]	0.59**	[0.47,0.75]	0.98	[0.75,1.28]
Cumulative number of HIV tests since age 14	0.88	[0.69,1.12]	1.01	[0.89,1.16]	1.04	[0.97,1.10]
						
"Risky" sexual partner						
Had an HIV test in last 6 months	3.54**	[1.48,8.45]	1.18	[0.33,4.16]	1.11	[0.61,2.01]
Cumulative number of HIV tests since age 14	0.55^†^	[0.27,1.11]	0.59	[0.25,1.40]	0.79^†^	[0.62,1.01]
						
Observations (person-months)	10617		13083		27351	

Note: We control for individual characteristics in the analyses

^†^p < 0.10, *p < 0.05, **p < 0.01

Overall, it appears that one-time HIV testing has little association with most measures of safe sexual behavior six months following the test for both males and females, and, where significant, it is associated with an increased likelihood of concurrent partnerships for males and engaging in "risky" and unprotected partnerships for never-pregnant females. However, repeated HIV testing appears to support safer sexual behavior, particularly decreasing the likelihood of concurrency among young males and "risky" sexual partnerships among both males and females. It is important to note that the associations that we uncover between repeat testing and safer sexual behaviors could also be due to unobserved heterogeneity, whereby those who choose to test repeatedly are more likely to be HIV negative and to display safer sexual behaviors to begin with.

## Discussion

This study utilized unique life history survey data collected using the Relationship History Calendar to examine the influence of the individual characteristics of youth and their recent sexual partnership histories on the likelihood of testing for HIV. The study also investigated the effect of HIV testing on subsequent sexual behavior. We found that over half of the respondents aged 18-24 years report at least one HIV test in the 10 years before the survey, which may reflect a trend of increased testing among youth, at least in western Kenya. This proportion is much higher than the proportion of Kenyan youth reporting ever undergoing an HIV test in the 2003 Kenyan Demographic and Health Survey [9] and is similar for females and higher for males to national testing estimates from the 2007 KAIS [8]. As stated previously, Kisumu has one of the highest HIV prevalence rates in the country. This has led to a mushrooming of HIV prevention programs in the area, and the high rate of testing may therefore partially reflect the increase in access.

Our study concentrated on the social context of uptake and how not only individual factors but also the characteristics of recent sexual partnership histories influence the decision to test for HIV. Similar to other studies [36], we found that urban residents were more likely to undergo an HIV test. Urban residence may reflect greater access to HIV information and services which indicates that greater attention may need to be paid on increasing this access in rural areas. With respect to sexual partnership history variables, marital aspirations were significantly associated with testing among females not pregnant at the time of first test, and having had unprotected sex in the previous six months was associated with testing for pregnant females, which likely reflects their pregnancy status. Young males with concurrent sexual partners in the previous six months were over twice as likely to be tested as those without concurrent partnerships. These results suggest that partnership characteristics and dynamics play a role in individuals' decision to test for HIV. Furthermore, while there is some evidence that young men involved in "risky" behavior are more likely to test, our results do not support the same for young women. As such, there may be need for gender- and relationship-specific interventions to assess risk and promote HIV testing.

We observed that non-pregnant females were approximately twice as likely to report an HIV test if they had a recent partner whom they wished to marry than if they did not wish to get married. This finding suggests that in a high-HIV context, as is the case in Kisumu, marital decisions may hinge on perceived exposure to HIV. Nationally, there is evidence of growing importance placed on HIV testing as a basis for entering a relationship. For example, in 11 out of 17 personal advertisements seeking relationship partners in a popular pullout magazine in one of the top national dailies in Kenya [37], there was reference to the HIV status of the seeker and/or desired status of the responder, or the advertisement stated that one of the requirements for entering the relationship was having an HIV test. As an illustration, typical advertisements might read: "...29, HIV +ve woman... is looking for a serious HIV +ve man aged between 30 and 40 for a long-term relationship leading to marriage..." or "...41, a tall HIV negative...man is looking for a mature lady aged between 30-40 for a relationship leading to marriage. She should be financially-stable, caring, loving, understanding, focused, God-fearing, and HIV negative." The increasing emphasis on HIV status as a basis for entering long-term relationships suggests that many individuals know their HIV status and expect their long-term partners to know theirs as well. Consequently, marital aspirations may drive the perceived need for a test. Marital aspirations also relate to more serious relationships within which condom use is less acceptable because it may be interpreted as a sign of mistrust, lack of acceptance, or infidelity [38]. Thus, the need for testing may be more salient for those who wish to marry because safe sex (in terms of condom use) is less likely to occur.

A larger proportion of females than males reported having had an HIV test, and 40% of females were pregnant when tested for the first time. PMTCT has been at the forefront of HIV/AIDS interventions in the country since 2001 when the national program was rolled out. Data from the 2007 KAIS show that 90% of women who gave birth between 2003-2007 attended an antenatal clinic (ANC) at least once and that 79% of all ANC attendees in 2007 received an HIV test [8]. Thus, based on our findings in Kisumu, it appears that promotion of HIV testing as part of PMTCT services may be gaining success. In addition, given the relatively high rate of pregnancy at first test for many young women, HIV testing among females may be mostly provider-driven rather than client-initiated. Thus, increased opportunities for provider-driven or routine testing for youth may enable the government to reach HIV testing coverage goals.

Motivations for HIV testing may affect its behavioral impacts and the effectiveness of testing on behavior change may be diminished for those who are tested because it is recommended (as part of PMTCT services) or required (e.g., for employment or insurance). However, our results suggests otherwise. We find that never-pregnant women (whose decision to test was more likely to be driven by their own concerns about their HIV status) were more likely to engage in "risky" sexual partnerships and practice unprotected sex in the six months after testing, while these associations were not observed among ever-pregnant women. Indeed, testing was associated with a decrease in unprotected sex among ever-pregnant women. Further research is needed to determine how and why behavior change is influenced by client- versus provider-initiated testing.

Previous studies of the impact of HIV testing on sexual behavior have found mixed results. We found limited evidence that HIV testing resulted in safe behavior six months after the test in terms of concurrency, the use of condoms, and engaging in "risky" partnerships. Indeed, we found that, in addition to increases in unsafe behaviors for never-pregnant females, HIV testing was associated with increased subsequent sexual concurrency for young males. In previous studies that observe a post-HIV test increase in the adoption of protective behavior [3,4], HIV counseling and testing was provided as part of an intervention, and therefore pre- and post-test counseling was standardized and of presumably high quality. In our study, we relied on self-reported data on the timing of HIV testing (which is likely to be in the absence of a direct HIV testing intervention). Further, we did not know whether the respondent received pre- and post-test counseling or its quality. Our findings, therefore, suggest that the short-term effects of generally available HIV testing and counseling may be weak, and further research into the characteristics of testing, including quality of testing available from public and private sources, is warranted to understand what intervening factors may affect the impact of testing on subsequent sexual behavior.

On a more positive note, we uncover some evidence that repeated testing may increase safe sexual behavior, specifically reducing the likelihood of engaging in "risky" types of partnerships for both males and never-pregnant females and concurrent sexual partnerships for males. These results were only marginally significant for some associations; however, they may indicate the potential benefits of repeated testing and counseling. While we postulate that repeat or routine testing may increase the adoption of safer sexual practices, we are cognizant that individuals who repeatedly get tested may be a select group that is in fact more likely to be HIV negative and less likely to engage in risk behavior. Further research on the characteristics of repeat testers that takes into account their HIV status would be helpful in enhancing our understanding of the drivers and impacts of repeat testing and its importance as an HIV/AIDS program strategy.

### Limitations

Our study findings must be interpreted in light of several limitations. First, the data were collected as part of a larger study focusing on the life course transitions and sexual relationship histories of youth. While details on sexual partnerships and changes over time allow us to examine more fully the social context and timing of HIV testing, details of attitudinal or other contextual factors that may affect testing, such as access to testing facilities [39], perceived risk for infection, or fear of HIV-related stigma [40], were not collected in the study. Second, ethical considerations did not allow us to enquire about the results of respondents' HIV tests. Although counseling is supposed to be offered regardless of positive or negative results and behavior change should hopefully take place in either event, other studies [5] have found differences in subsequent behaviors by HIV status. Finally, although we adopted the use of a calendar to enhance recollection of past events and decrease social desireability bias, the data are still subject to other types of recall and self-reporting biases.

## Conclusions

Overall, given young people's susceptibility to HIV, we highlight key factors that may be important determinants of HIV testing among this young population in a high HIV prevalence setting in sub-Saharan Africa. Our findings point to the potential success of PMTCT programs in increasing the uptake of HIV testing among pregnant females. In light of this, we suggest that provider-driven or routine testing may be an important alternative to voluntary counseling and testing in increasing HIV testing among youth. Further research on the potential for school- or home-based testing in increasing the number of young people who are aware of their HIV status may be warranted in this respect. Consistent with past research, we find that urban residence is associated with a greater likelihood of testing. As such, there may be need to ensure that rural residents are not marginalized in the provision of HIV information and testing services. Although we find little evidence supporting the impact of HIV testing on unsafe sexual behavior, our results suggest that repeat testing may be associated with the adoption of safer behaviors. Again, encouraging repeat testing (and counseling) as part of routine health care appears to be one avenue to reduce the prevalence of unsafe sexual practices. Finally, our data on partnership characteristics suggests that marital aspirations may be an important driver of HIV testing among females. Given trends showing an increasing emphasis on HIV status as a basis for entering long-term relationships, couples testing may be an important means to increase testing uptake.

## Competing interests

The authors declare that they have no competing interests.

## Authors' contributions

CWK conceptualized the manuscript idea, conducted the data analyses, participated in the literature review, and prepared the first draft of the manuscript. NL was the primary investigator for the larger study from which the paper is drawn, made substantive contribution to the conceptualization of the manuscript, reviewed the literature, informed the data analyses, and assisted with revising the manuscript. COI and EMZ made substantive contributions to the conceptualization of the manuscript. All authors critically reviewed the manuscript. All authors are aware that the manuscript is being submitted to the journal.

## Pre-publication history

The pre-publication history for this paper can be accessed here:

http://www.biomedcentral.com/1471-2458/10/412/prepub
